# Flexible high-density microelectrode arrays for closed-loop brain–machine interfaces: a review

**DOI:** 10.3389/fnins.2024.1348434

**Published:** 2024-04-15

**Authors:** Xiang Liu, Yan Gong, Zebin Jiang, Trevor Stevens, Wen Li

**Affiliations:** ^1^Neuroscience Program, Department of Physiology, Michigan State University, East Lansing, MI, United States; ^2^Institute for Quantitative Health Science and Engineering (IQ), East Lansing, MI, United States; ^3^Department of Electrical and Computer Engineering, Michigan State University, East Lansing, MI, United States; ^4^Department of Biomedical Engineering, Michigan State University, East Lansing, MI, United States

**Keywords:** microelectrode array, closed-loop, brain-machine interfaces, flexibility, high density

## Abstract

Flexible high-density microelectrode arrays (HDMEAs) are emerging as a key component in closed-loop brain–machine interfaces (BMIs), providing high-resolution functionality for recording, stimulation, or both. The flexibility of these arrays provides advantages over rigid ones, such as reduced mismatch between interface and tissue, resilience to micromotion, and sustained long-term performance. This review summarizes the recent developments and applications of flexible HDMEAs in closed-loop BMI systems. It delves into the various challenges encountered in the development of ideal flexible HDMEAs for closed-loop BMI systems and highlights the latest methodologies and breakthroughs to address these challenges. These insights could be instrumental in guiding the creation of future generations of flexible HDMEAs, specifically tailored for use in closed-loop BMIs. The review thoroughly explores both the current state and prospects of these advanced arrays, emphasizing their potential in enhancing BMI technology.

## 1 Introduction

Brain-machine interfaces (BMI) have emerged as a powerful tool for understanding the brain (Janjua et al., 2021), restoring lost function in individuals with neurological disorders (Johnson et al., 2016; Mohammed et al., 2022) and enhancing brain functions (Olsen et al., 2022). Closed-loop BMIs, in which neural activity is recorded and decoded in real-time to drive external devices or stimulate neural tissue, hold promise for enhancing the accuracy and efficacy of BMI applications (Tanskanen et al., 2020). These systems are widely utilized in neurorehabilitation (Ang et al., 2015; Donati et al., 2016; Liu and Ushiba, 2022), prosthetics control (Ajiboye et al., 2017), treatment of severe neurological disorders (Ouyang et al., 2023), and adaptive deep brain stimulation (Castrioto and Moro, 2013; Little et al., 2016; Scholten et al., 2020), among other fields. Typically, neural signals (e.g., electrical activity of neurons) are initially recorded using a neural interface device, such as a microelectrode array (MEA). These signals are then decoded and processed to derive user intentions, which are translated into commands for controlling an external device. Subsequently, feedback from the device, often in the form of electrical stimulation, is delivered back to the brain, closing the loop (Zhang et al., 2023). This contrasts with open-loop systems, which might only read brain signals or stimulate the brain without any real-time feedback (Tanskanen et al., 2020).

A MEA consists of a grid of tightly spaced microelectrodes that can be used to detect electrophysiological signals from neurons or to deliver electrical pulses to stimulate neural activity (Adams et al., 2005). MEAs are commonly integral to closed-loop BMIs, serving as components that either decode neural signals, deliver electrical stimulation back to the brain, or perform both functions, thus enabling bidirectional communications. Conventional MEAs with single channel or multiple channels separated with far distance(>200 μm) have historically played a vital role in the development and applications of closed-loop BMIs (Chou et al., 2011). However, the high-density microelectrode arrays (HDMEAs), with significantly enhanced density and channel count arranged on a limited area (Lee et al., 2022), offer several advantages compared to conventional MEAs. First, HDMEAs can detect and stimulate neural activity with higher spatial resolution and greater precision, enabling more detailed mapping of neural networks and a better understanding of complex brain functions. Second, the high density of electrodes provides a more comprehensive dataset. This richness of data is essential for advanced neural decoding algorithms, which can translate neural activity into more accurate and nuanced commands for BMIs. Third, in lower-density arrays, signals from multiple neurons are often averaged over larger electrode areas, potentially losing critical information. HDMEAs can reduce this spatial averaging, capturing more discrete signals from individual or small groups of neurons. Finally, with more electrodes interacting with neurons, HDMEAs can potentially facilitate more complex and nuanced interactions between the BMI and the brain, leading to more sophisticated applications and therapies.

HDMEAs can be categorized into rigid and flexible types, depending on the material of the substrate used for fabricating the microelectrodes (Tanwar et al., 2022). At present, the most advanced HDMEAs are rigid leveraging the state-of-the-art silicon manufacturing technology. For example, Neuropixels 2.0, a miniaturized high-density silicone-based probe, has total of 1,280 channels on one shank with a profile of 10 mm × 70 μm × 24 μm (Steinmetz et al., 2021). However, rigid HDMEAs often encounter limitations (Johnson et al., 2016; Kolaya and Firestein, 2021; Oldroyd and Malliaras, 2022), including inflammatory response, tissue damage, limited long-term stability, and biocompatibility issues.

Flexible MEAs, often fabricated from soft materials like polyimide or parylene, can adeptly mold to the neural tissue and provide less mismatch between interfaces and brain tissue, reducing the risk of tissue damage and ensuring a more stable, intimate interaction with neurons (Araki et al., 2020). Furthermore, the adaptability of flexible MEAs allows them to accommodate physiological changes and movements within the brain, maintaining consistent interfacing and thereby supporting the longevity and robustness of the BMI system (Sharafkhani et al., 2022; Xu et al., 2022). Consequently, flexibility in MEAs is not merely a beneficial attribute but a quintessential one, ensuring the viability, reliability, and efficacy of closed-loop BMIs in both research and clinical applications. Further merging the benefits of both, flexible HDMEAs bring the adaptability of flexible substrates to high-resolution electrode arrays, proving invaluable for discerning intricate neuronal dynamics, especially in dynamic or soft tissue environments where precision and conformability are paramount (Zhou et al., 2023).

Apart from flexibility and high density, the ideal profile of flexible HDMEAs for closed-loop BMIs comprises several crucial attributes. These include biocompatibility (Shepherd et al., 2021), high-quality performance (Nam, 2021), and long-term stability (Ward et al., 2009; Golabchi et al., 2019), etc. However, the development of flexible HDMEAs with these ideal properties entails confronting both the general challenges inherent to HDMEAs and specific challenges unique to their flexible design, encompassing mechanical (Oldroyd and Malliaras, 2022), electrical (Szostak et al., 2017; Qiang et al., 2021), biological (Golabchi et al., 2019), chemical (Shepherd et al., 2021), and interconnection issues (Behfar et al., 2021). In response, researchers employ strategies spanning material exploration (Bonafe, 2022; Chik et al., 2022), flexible HDMEA design innovation, advanced fabrication strategies (Scholten et al., 2020; Shah et al., 2022; Xiang et al., 2022), engineering optimization (Chik et al., 2022; Yan et al., 2022; Yin et al., 2022), and holistic combination strategies (Eunah et al., 2022; Hong et al., 2022), aiming to enhance performance and minimize risks in flexible neural interfacing devices.

The aim of this review is to provide a comprehensive examination of flexible HDMEAs within the realm of closed-loop BMIs. It offers a detailed analysis of what constitutes high-density configuration, the core principles of HDMEA design and fabrication, alongside showcasing the latest exemplars in the field. The review critically identifies and discusses the spectrum of challenges that are currently faced including mechanical, electrical, chemical, biological, and those related to high-density interconnections. Subsequently, it discusses innovative strategies and recent advancements in materials, design, surface modifications, and fabrication that are paving the way for the next generation of flexible HDMEAs. The ultimate goal is to elucidate the trajectory of flexible HDMEAs’ evolution, framing the current state while projecting into the future implications for closed-loop BMIs.

## 2 Flexible HDMEAs in closed-loop BMIs

### 2.1 Definition of high density

For a long time, we lacked a clear definition of HDMEAs. Among the published studies, MEAs that labeled high density have electrode densities ranging from less than one channel per square millimeter to thousands of channels per square millimeter. The variability in HDMEA definitions can be attributed to application-specific density requirements—high for brain neural spaces under 100 μm and lower for muscular targets over several thousand square microns (Wiedemann and McDaid, 2017; Choi et al., 2020)—and the diversity in materials and technologies, which results in differing channel densities, ranging from a few to thousands of channels per square millimeter. In a recent paper, the high density was defined as the spacing among electrode sites being no more than 100 μm (Wang et al., 2023). Considering our discussion on flexible HDMEAs is focused on BMIs that targets neurons in brain tissue in this article, we mainly select flexible HDMEAs that have a pitch within 200 μm.

### 2.2 Design and fabrication principles of flexible HDMEAs

The fabrication of flexible HDMEAs requires a comprehensive strategy that seamlessly integrates device geometries, material selection, fabrication techniques and approaches, surface modification and packaging methods, etc. Key considerations in fabrication of flexible HDMEAs are discussed below.

**Figure 1:**
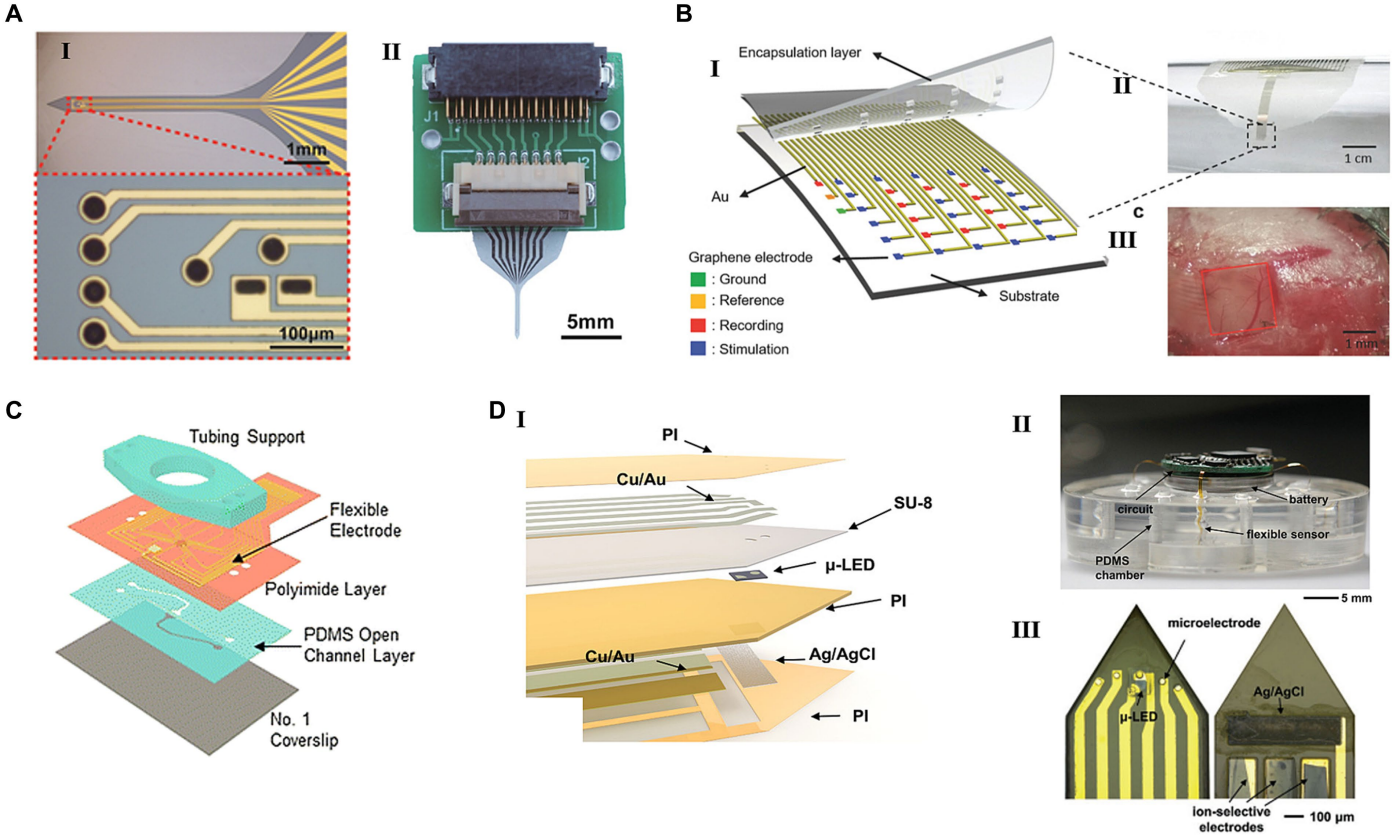
Flexible HDMEAs integrated with different stimulation modules. **(A)** Flexible HDMEAs probe with electrical stimulus and neuronal firings detection. (I) The image of the neural probe’s shank and the pattern of the microelectrodes. (II) The fully fabricated probe connected to a customized PCB connector. Reproduced from Chik et al. (2022) with permission from 2022 Wiley-VCH. **(B)** Flexible graphene MEA with electrical stimulation for epilepsy. (I) Schematic of a graphene-based seizure sensor. (II) Optical images of an epilepsy treatment sensor on a tube. (III) Photograph of a 30-electrode transparent array on the mouse brain. Reproduced from Park et al. (2018) with permission from 2018 WILEY-VCH. **(C)** Microfluidic perforated MEAs with the capability of locally delivering chemical stimulation. Reproduced from Esteban-Linares et al. (2023) with permission from 2023 The Royal Society of Chemistry. **(D)** Flexible electronics generating optical and electrical stimulation in multi-Encephalic Regions. (I) The exploded view of a single flexible channel that contains a μ-LED, four microelectrodes, and three ion-selective sensors. (II) An image of a wireless circuit connected with four channels that have been inserted into a PDMS chamber. (III) Images of the top and bottom sides of an implanted channel. Reproduced from Ling et al. (2020) with permission from 2020 WILEY-VCH.

**Figure 2:**
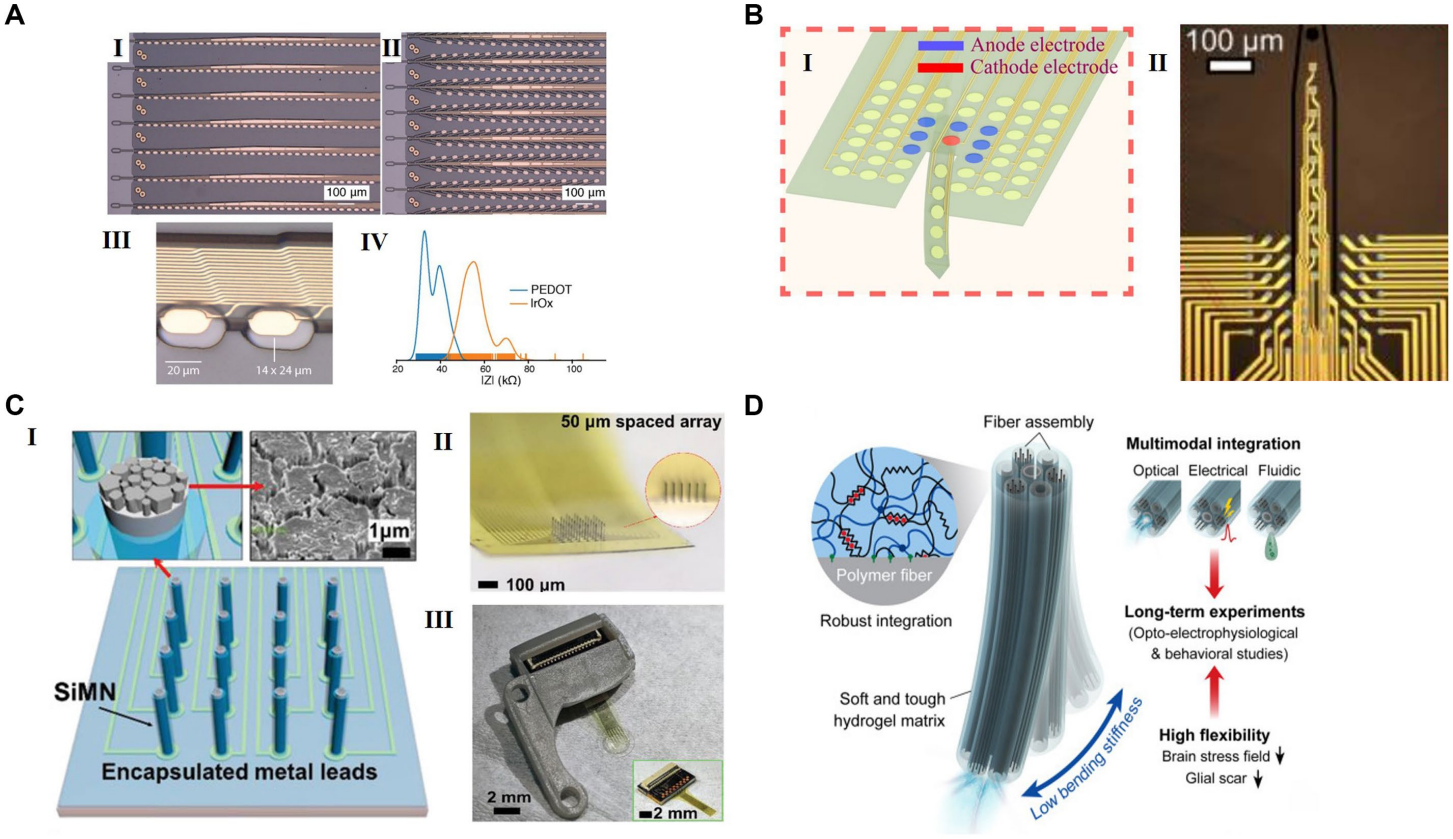
Representative flexible HDMEAs in various configurations for use in closed loop. (A) Multi flexible electrode “threads” with as many as 3072 electrodes per array distributed across 96 threads. (I) A packaged sensor device. (II) Image of individual electrodes for the thread design in panel I. (III) “Linear Edge” probes, with 32 electrode contacts spaced by 50 μm. (IV) “Tree” probes with 32 electrode contacts spaced by 75 μm. Reproduced from Musk (2019), licensed under CC BY-ND 4.0. (B) Schematic of optically transparent surface arrays with an insertion probe(I) and the micrograph(II). Reproduced from Uguz and Shepard (2022), licensed under CC BY NC 4.0. (C) A scalable silicon microneedle array (SiMNA) on thin, transparent, and flexible substrates. (I) A schematic of a 32-channel SiMNA on flex with polyimide passivating the metal leads and parylene-C passivating the SiMNA (excluding the tips). Inset on the left shows magnified view of the exposed SiMN tip with PtNM coated on Si, and the inset on the right shows the scanning electron microscopy (SEM) image of the PtNM surface at the tip. (II) A magnified optical image of 32-channel SiMNA with 50 μm needle-to-needle spacing. The inset shows the magnified photograph taken from the side of the SiMNA. (III) A photograph of the device mounted on 3D-printed custom headpost. Reproduced from Lee et al. (2022) with permission from 2022 Wiley-VCH. (D) Neural probes with adaptive bending stiffness determined by the hydration states of the hydrogel matrix. Rproduced from Park et al. (2021) with permission from 2022 The Authors.

1. Device geometries of a HDMEA encompass the spatial and physical configurations, including the arrangement, shape, size, and spacing of the electrodes, along with the overall structure of the device. This involves the meticulous layout of electrodes, which can vary in shape and size, and are strategically spaced to capture localized neural activity effectively. The shape and spatial configuration of HDMEAs are crucial for neural interfacing, with designs ranging from flat, planar structures (Zeng et al., 2022) to complex, curved (Ji et al., 2020a), or three-dimensional (3D) forms (Le Floch et al., 2022; Brown et al., 2023; McDonald et al., 2023) tailored to specific anatomical contexts, such as the cortical surface or deep brain regions. The overall shape impacts the HDMEA’s ability to establish a stable interface with neural tissues, its mechanical stability, and its long-term biocompatibility, while the arrangement of electrodes—potentially organized in grids (Park et al., 2018; Figure 1B), linear arrays (Musk, 2019; Zhou et al., 2023; Figures 2B,C, 4A)—and their individual shapes and sizes are meticulously crafted to enhance signal acquisition and stimulation capabilities.
2. The selection of electrode materials, encompassing not only the substrate but also the conductive elements, insulating materials, and materials utilized in other components, holds critical importance in the development of flexible HDMEAs. For substrates in neural interfaces, the most commonly used materials encompass polymers, such as polyimide (Schander et al., 2021; Steins et al., 2022), polydimethylsiloxane (PDMS) (Schiavone et al., 2020; Chong, 2022), parylene C (Golda-Cepa et al., 2020; Wang et al., 2020), as well as other materials such as silk (Cointe et al., 2022), medical adhesive tape (David-Pur et al., 2014), hydrogels (Zeng and Wu, 2022), predominantly chosen for their biocompatibility, flexibility, and electrical insulation, ensuring safe and stable interfacing with neural tissues. An ideal electrode material needs to meet at least the following requirements: (1) compatible with cell/tissue without causing any changes in physiology and viability, (2) have good conductivity and low impedance to ensure a high signal-to-noise ratio (SNR), (3) have stable mechanical, physical, and chemical properties in the physiological environment (Zhu et al., 2021). The selection of substrate materials is usually based on the properties such as electrical insulation, biocompatibility, durability, transmittance, etc. The most widely used electrode materials are inert metals, such as gold (Au), platinum (Pt), platinum black, iridium (Ir), and iridium oxide (IrOx), renowned for their high conductivity and biocompatibility, but many promising new materials have been developed for various needs in recent years.
3. The development of flexible HDMEAs also necessitates careful consideration of fabrication methods, surface modification techniques, and packaging strategies to ensure optimal performance and durability in neural interfacing applications. Fabrication methods for Flexible HDMEAs intertwine various techniques to construct devices tailored to specific neural interfacing applications, with conventional MEAs often leveraging photolithography, etching, and physical vapor deposition to meticulously pattern and construct electrodes on typically rigid substrates like silicon. Surface modification of flexible HDMEA includes changes in surface chemicals and morphology, which can not only affect the surface area and change the impedance to improve SNR (Szostak et al., 2017; Niederhoffer et al., 2023), but also facilitate cellular adhesion of neurons to the surface of flexible HDMEAs (Brown et al., 2023). Besides, packaging strategies are crucial to the reliability and lifetime of flexible HDMEA (Kim et al., 2012; Seok, 2021; Shen and Maharbiz, 2021). Suitable packaging should ensure the hermeticity for HDMEAs, to protect the inner parts of the HDMEAs from the physical environment, and to protect the host from any injury caused by chemical leakage or electrical leakage (Seok, 2021; Yang et al., 2021a,b).

**Figure 3:**
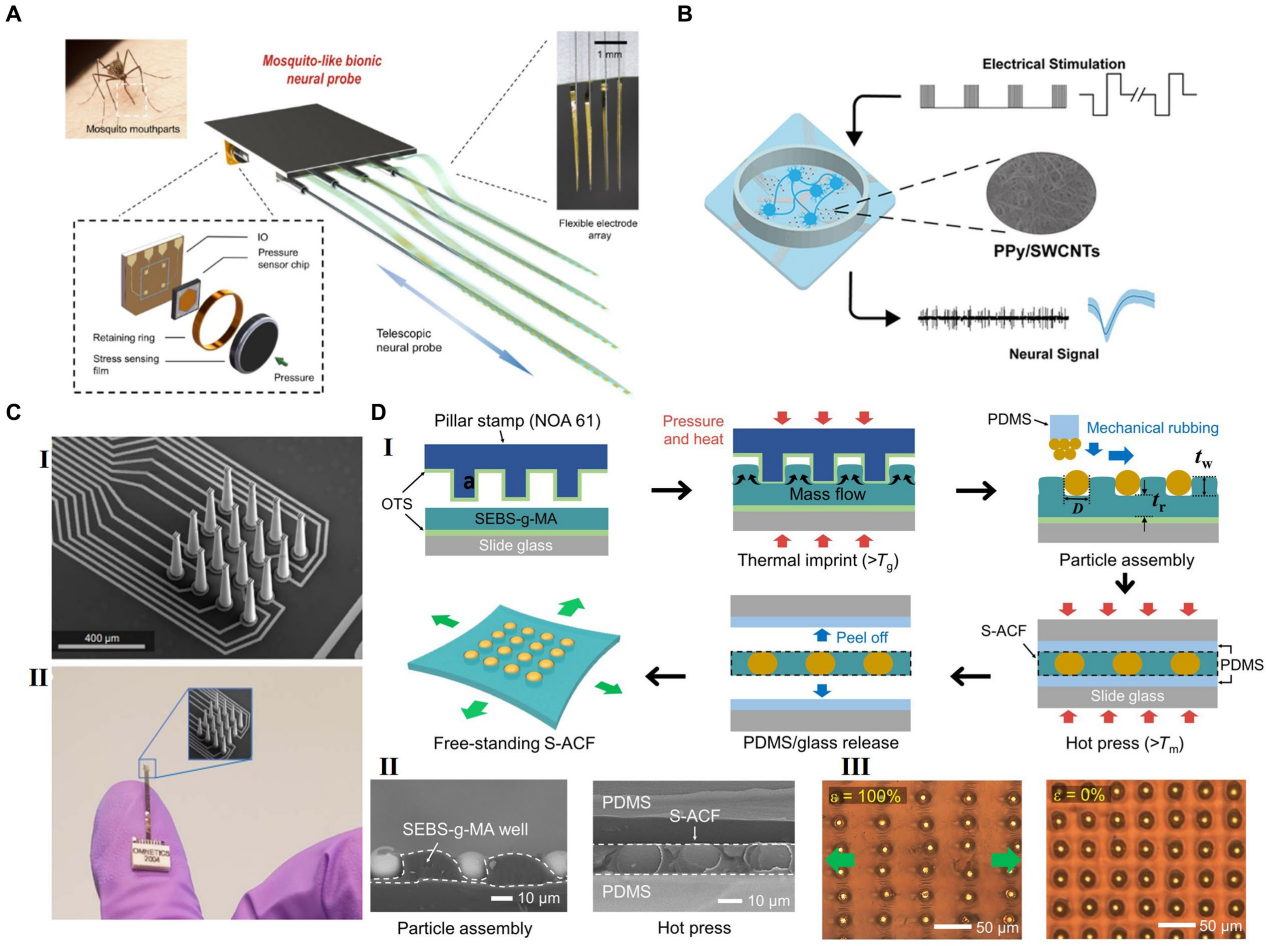
Strategies for future flexible HDMEAs. (A) Multifunctional biomimetic neural probe system, with multichannel flexible electrode array and high sensitivity sensor array. Adapted from Zhou et al. (2023), licensed under CC BY 4.0. (B) PPy/SWCNTs-modification on microelectrodes for electrical stimulation and detection. Reprinted with permission from Yang et al. (2023). Copyright 2023 American Chemical Society. (C) 3D-printed arrays for neural recording. Adapted with permission from Brown et al. (2023), licensed under CC BY 4.0. (D) A periodic arrangement of stretchable ACF (S-ACF) incorporated within a thermoplastic block copolymer film. (I) Scheme for the fabrication of the S-ACF. (II) Left: Cross-sectional SEM image taken after the particle assembly in the template. Right: Cross-sectional SEM image of the S-ACF after the hot pressing. (III) Optical images of the S-ACF when stretched (ε = 100%) and strain released (ε = 0%). Adapted with permission from Hwang et al. (2021), licensed under CC BY 4.0.

**Figure 4:**
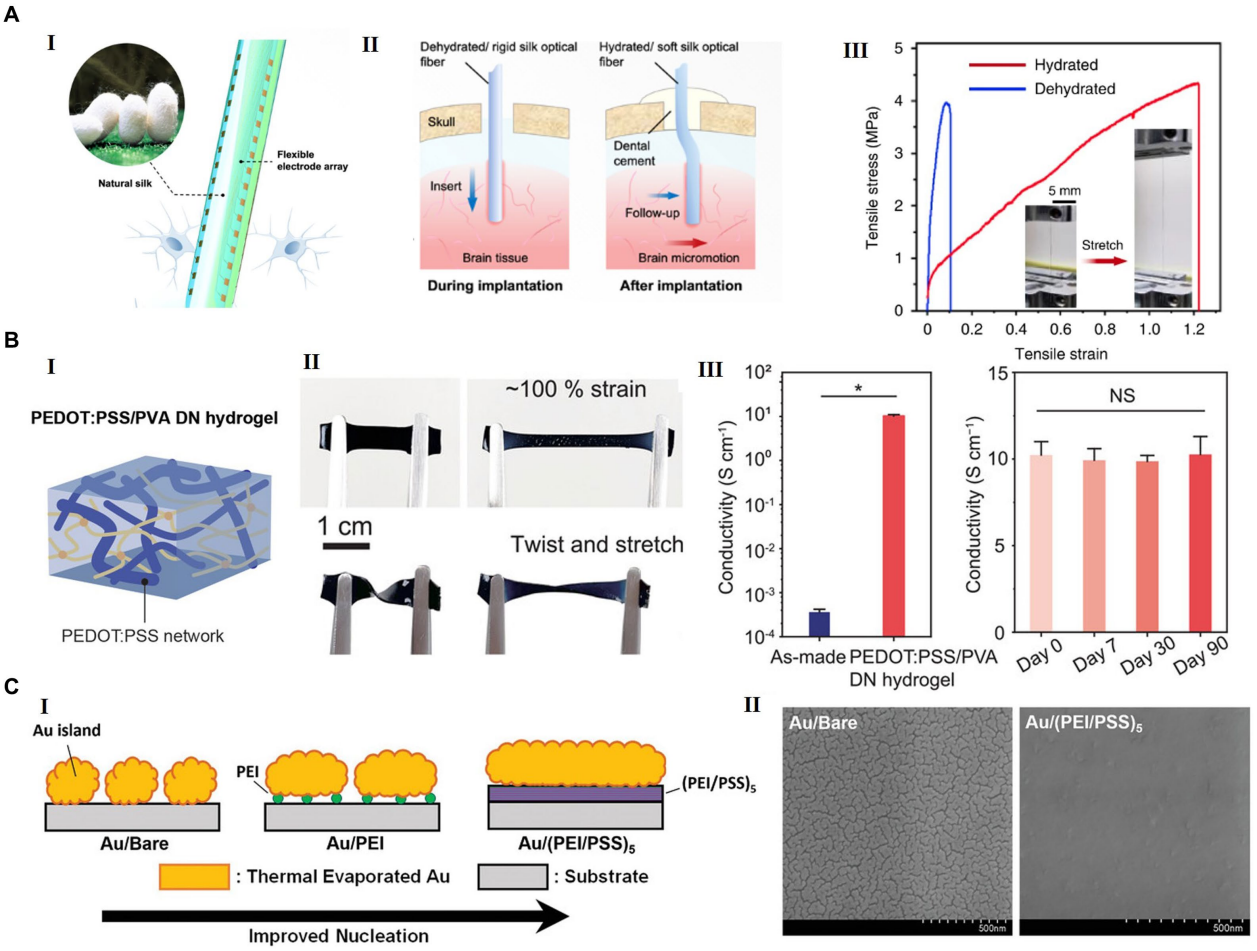
Examples of advanced materials for future flexible HDMEAs. (A) Silk-Optrode comprising a silk protein optical fiber substrate and multiple flexible HDMEAs. (I) A schematic of a silk optical fiber-based Silk-Optrode probe and the multilayer structure of the device. (II) A schematic of the probe implanted into brain tissue, comparing with the rigid probe and the flexible silk optical fiber. (III) The strain–stress curve of the silk optical fiber in the hydrated and dehydrated states. Inset, typical hydrated silk optical fiber before and after stretching. Reproduced from Zhou et al. (2022), licensed under CC BY 4.0. (B) Highly conducting and stretchable double-network hydrogel. (I) Schematic of PEDOT:PSS/PVA double network(DN) hydrogel. (II) PEDOT:PSS/PVA hydrogel under stretching, twisting, and twisted stretching. (III) Electrical conductivity of the as-made hydrogel and the PEDOT:PSS/PVA DN hydrogel with acid treatment and conductivity of the PEDOT:PSS/PVA DN hydrogel for 3 months. Reproduced from Li et al. (2022) with permission from 2022 Wiley-VCH. (C) A new structure of transparent and flexible MEA, with ultrathin gold nanofilm with polyelectrolyte multilayers (PEM) as the metallic film nucleation-inducing seed layer (NISL). (I) Schematic diagrams of the formation of ultrathin gold electrodes on polyelectrolyte coatings. (II) SEM images of 8 nm-thick gold electrodes deposited on a bare silicon substrate (left) and a (PEI/PSS)5 NISL coated on silicon (right). Reproduced from Hong et al. (2022) adapted with permission from 2021 Wiley-VCH.

### 2.3 Examples of state-of-the-art flexible HDMEAs for closed-loop BMI

This section highlights the latest advancements in flexible HDMEAs for closed loop BMIs over the past five years. The summary of the most recent flexible HDMEAs integrated in closed-loop BMIs is shown in Table 1.

**Table 1:** Summary of the most recent flexible HDMEAs integrated in closed loop BMIs.

References	Electrodes numbers	Electrode size	Center to center distance	Electrode materials	Packaging materials	Lifetime being tested
Guo et al. (2021)	16	Dia = 20 μm, 25 μm, 30 μm	150 μm	Cr, Au	Parylene	28 days
Vajrala et al. (2023)	4	Dia = 40 μm, 100 μm	100 μm*	Ti, Au	Parylene	8 weeks
Kauth et al. (2024)	30	1,910 μm^2^	50.9 μm	Ti, Au	Polyimide	24 h
Zhou et al. (2023)	128	N/A	N/A	Au	Polyimide	16 weeks
Chik et al. (2022)	8	20 × 40 μm	50 μm	Au	Parylene	5 weeks
Max et al.(2022)	60	Dia = 10 μm, 22 μm, 50 μm, 150 μm, 582 μm, 1,000 μm	200 μm	Indium tin oxide	Polyimide	N/A
Wang et al. (2023)	8	Dia =25 μm	70 μm	Cr, Au	Polyimide	N/A
Zachariah et al. (2021)	60	100 μm^2^	40 μm	Platinum	Polyimide	6 weeks
Ling et al. (2020)	16	Dia = 30 μm*	100 μm*	Au	Polyimide	2 weeks
Silveira et al. (2020)	24	25 μm × 50/70 μm	50/70 μm	Ti	Parylene	N/A
Guo et al. (2021)	8	Dia = 25 μm	70 μm	Cr/Au	Polyimide	N/A
Yu et al. (2021)	32	30 μm × 30 μm	200 μm*	Cu	Polyimide	N/A
Wang et al. (2023)	8	Dia = 25 μm	70 μm	Cr/Au	Polyimide	N/A
Uguz and Shepard (2022)	60	Dia = 15 μm	40 μm	PEDOT:PSS	Parylene	20 days
Seo et al. (2020)	20	Dia = 150 um	350 μm	Ti, Au	PDMS, Parylene-C	N/A
Esteban-Linares et al. (2023)	16	30 × 30 and 40 × 40 μm^2^ for graphene, and 60 × 60 and 80 × 80 μm^2^ for Au	200 μm	Graphene or Ti/Au	Polyimide on PDMS	N/A
Yang et al. (2021a,b)	128	Dia = 30 μm	50 μm	PEDOT:PSS	Parylene C	N/A

* The value comes from an estimate of the pictures provided in the original articles.

Neuralink introduced a scalable high-bandwidth BMI system. This system includes arrays of 96 flexible polymer threads, each with 32 electrodes, yielding a total of 3,072 electrodes (Musk, 2019; Shown in Figure 2A). The electrodes are made of gold and treated with surface modifications by using the electrically conductive polymer poly-ethylenedioxythiophene doped with polystyrene sulfonate (PEDOT: PSS) and IrOx, in order to lower the impedance for electrophysiology and increase the effective charge-carrying capacity of the interface. The electrode array is packaged with polyimide into a compact implantable device with custom chips for low-power amplification and digitization. The entire package for 3,072 channels is less than 23 × 18.5 × 2 mm^3^. The thin-film HDMEAs provide superior brain tissue compatibility and precise, robot-assisted placement for targeted brain mapping. Their compact design integrates a high-channel-count application specific integrated circuit (ASIC), optimizing size and power efficiency.

Uguz and Shepard (2022) presented a high-density, mechanically flexible subdural surface microelectrode array for spatially controlled, bipolar, cortical stimulation (Shown in Figure 2B). The electrodes are made of gold, coated with PEDOT: PSS, and packaged by an ultrathin film (4 μm) parylene. These electrodes have a diameter of 15 μm and pitch of 40 μm. with high-capacitance (>1 nF) and single neuronal resolution, the electrodes can be programmed to shape the charge injection front selectively at depths approaching 300 μm with a lateral resolution better than 100 μm. To combine surface stimulation with electrical depth recording, the flexible HDMEA incorporates an implantable shank on the same substrate. The shank extension has 10 electrodes reaching down to 500 μm below the pial surface.

Zhou et al. (2023) developed a neuro-probe system, mimicking mosquito mouthparts, with four shanks, each housing 32-channel electrodes, totaling 128 channels (Figure 3A). Polyimide was used as its insulation layer, and metal stack of 5 nm chromium/150 nm nickel/50 nm gold was used as conductive layer. The flexible HDMEAs are 2.5 μm thick and 105 μm wide. The electrodes measure 25 × 15 μm^2^ in size and are spaced 70 μm apart. These arrays were mounted on a sharpen tungsten wire to facilitate the insertion process. Each of the shanks could be inserted into the brain independently to target different regions. The system also has sensitive sensors to monitor insertion pressure without visual aid. It offers early warning detection for delicate intracranial tissues like vessels, minimizing the risk of damage during implantation. The system demonstrates outstanding postoperative performance, with a unit yield of up to 57% recorded 12 h after surgery, and 77% of unit activities being tracked from 4 to 12 weeks.

## 3 Challenges in developing and applying flexible HDMEAs in closed-loop BMIs

Although the aforementioned state-of-the-art flexible HDMEAs have advanced the field in various aspects, the development of an ideal flexible HDMEA still requires further improvements. For instance, in terms of density, Neuralink has increased the electrode count to 3,092 through multiple threads (Musk, 2019), yet the density per thread still significantly lags behind the most advanced rigid-based HDMEAs. Moreover, the insertion of flexible electrodes remains a significant challenge. Although robots (Musk, 2019) and specially designed insertion tools like mosquito mimic needles (Zhou et al., 2023) can enhance insertion precision and reduce damage, these auxiliary methods, despite their improvements, cannot completely eliminate the direct brain tissue damage caused by the insertion assistants. Additionally, implants designed for human brain applications are expected to be reliable over long periods, potentially spanning years, but research into their long-term stability is not yet exhaustive. In essence, the development of flexible HDMEAs encounters several common challenges, and the creation of an ideal flexible HDMEA involves addressing both the general challenges inherent to HDMEAs and specific challenges unique to their flexible design. These challenges encompass mechanical, electrical, biological, chemical, and interconnection issues, among others, which will be thoroughly discussed in the following text.

### 3.1 Mechanical challenges

Except the general mechanical failures for MEAs, flexible HDMEAs present unique mechanical challenges rooted in their respective material and structural properties. The main failures of flexible HDMEAs in mechanical aspect could be classified into three types: (1) intrinsic failure of stable function, (2) insertion failure, and (3) failure to long time use in physiological environments.

The intrinsic failure of stability function emerges from the very nature of the flexible HDMEA, regardless of its operational environment. Typical problems include physical fracture or cracking of the electrodes, damage to the metal layer via corrosion, delamination due to poor adhesion between layers, the failure of the insulation layer led by pinholes or water penetration (Oldroyd and Malliaras, 2022), thereby preventing the development of flexible HDMEAs.

The insertion of flexible HDMEAs into biological tissues introduces a substantial challenge (Scholten et al., 2020); maintaining vital flexibility, and/or minimizing cross-sectional dimensions might undermine stiffness, potentially jeopardizing the requisite rigidity for accurate insertion and inadvertently causing microelectrode buckling, tissue damage, or electrode misplacement (Sharafkhani et al., 2022). The challenge arises if the stiffness of flexible devices is insufficient for neural tissue penetration, which often resulting in a more complex and less preferable insertion procedure (Shi et al., 2020; Xu et al., 2022).

The harsh biological setting where electrodes are placed can compromise their mechanical stability, especially long-term post-implantation (Oldroyd and Malliaras, 2022; Ramesh et al., 2022). This environment can induce electrode fracture, corrosion, delamination, swelling, dissolution, and mechanical stress, notably in the presence of continuous electrical stimulation (Oldroyd and Malliaras, 2022).

### 3.2 Electrical challenges

Developing HDMEAs suffers from high initial impedance because of the small-feature geometry, so that smaller electrodes are inherently noisier, exhibit worse recording quality, and are less functional because of decreased maximum possible stimulating current (Szostak et al., 2017). The rational impedance magnitude of electrodes at 1 kHz will be needed for quality recording. At least 40–150 kΩ is necessary in order to achieve the selective detection of the action potential from a single unit, but electrode’s impedance greater than 5 MΩ recording of neural signals is overpowered (Buzsáki, 2004; Prasad and Sanchez, 2012).

Even though the electrodes implanted into tissues for recording performed well in the acute phase, the performance was inconsistent and even lost the recording ability over a longer period (Merrill and Tresco, 2005). Impedance will increase as diffusional barrier is formed in the body (Merrill and Tresco, 2005; Abidian et al., 2010). Encapsulation failure and electrode breakage are another issue that might happen over time. A long-term *in vivo* study indicated that failure of the insulation material is the most significant factor in the reduction of both signal quality and impedance of implanted electrodes (Barrese et al., 2013).

Crosstalk is another limitation of high-density recording devices, which may occur between electrodes if the space is too close, resulting in low SNR, signal transmission error, or signal loss (McNamara et al., 2021; Qiang et al., 2021). For neural applications, the crosstalk level has to be below 1% of the recorded signal level to make it negligible compared with the background noise (Najafi et al., 1990). The electrode spacing should also be well designed, in which HDMEAs work in an excellent spatial solution with minimum crosstalk between electrodes (Cruz et al., 2019).

In neural stimulation, the perfect electrode requires a robust storage capacity to seamlessly transmit current pulses, simultaneously curbing potential variations at the electrode/tissue nexus, thereby mitigating electrode polarization and thermal accumulation (Suresh Vajrala et al., 2021), which is a task that’s notably arduous for flexible HDMEAs.

### 3.3 High-density interconnection challenges

Apart from the neural interface device itself, there’s an inconspicuous yet critically important technical challenge: the integration of flexible sensor and backend stage (Behfar et al., 2021). This unique challenge restricts system integration and directly affects the stability and performance of HDMEAs (Luo et al., 2023). SNR and low latency can potentially be improved through advancements in algorithms and backend technologies (Boran et al., 2019; Siddiqui et al., 2019; RaviPrakash et al., 2020), but the wiring issue is the true critical problem that deeply troubles HDMEA designers. Traditional silicon-based probes, benefiting from the development of modern electronics, have various mature and stable connection methods. However, the flexibility of the HDMEAs itself becomes a challenge for stable connections with subsequent systems (Jang et al., 2022; Miyamoto et al., 2022). Setting stability aside, the hundreds or even thousands of connection points on HDMEAs make manual connections extremely difficult without specific tools or methods. This also restricts the possibility of expanding more electrodes on flexible HDMEAs (Tchoe et al., 2022). The reliability issue of the soft-hard interface presents an additional challenge for making high density interconnection between flexible HDMEAs and backend electronics. The instability caused by inconsistent stress/strain concentration at the interface of two mechanically dissimilar materials can lead to delamination failure.

### 3.4 Chemical challenges

In the existing literature, comprehensive reviews (Oldroyd and Malliaras, 2022; Zeng and Huang, 2023; Ziai et al., 2023) discussed major chemical challenges of developing and applying MEAs in biological circumstances. Here we provide a concise overview. The challenges posed by the chemical environment to flexible HDMEAs are multifaceted, which include (1) the effects of the physical chemicals on electrodes, (2) the effects of the chemicals from the HDMEAs to the host tissues, and (3) stability for the long-term use. The physical intricate mix of organic and inorganic chemicals can impact the performance and longevity of the flexible HDMEAs. Despite the diverse materials and methods used in the fabrication of MEAs, many are not adequately prepared to handle the demanding physiological conditions. The physical chemical environment can threaten the stability of MEAs over both short and long durations, owing to a range of organic and inorganic reactions.

Additionally, the electrode’s inherent chemical instability can be potentially harmful to the organism. Pt, for instance, has long been regarded as highly biocompatible and non-toxic. However, a recent study by Shepherd et al. (2021) revealed that prolonged stimulation (lasting 6 months with a charge density of 267 μC/cm^2^/phase) of Pt electrode arrays led to a notable production of Pt particulates. This, in turn, resulted in an escalated fibrous tissue response and weakened the quality of long-term records.

### 3.5 Biological challenges

Biocompatibility is a crucial criterion for materials utilized in biosensors, ensuring they integrate harmoniously within biological environments. Yet, even when employing these biocompatible materials, inherent biological challenges continue to arise and need addressing (Otte et al., 2022). Risks that can cause damage to the tissue and induce an acute inflammatory reaction include direct damage during surgery, mismatches between the electrode and tissue, micromovements of the electrodes, damage by released chemical from the electrodes, etc. (Kozai et al., 2015). The body’s responses to implanted HDMEAs include acute (Polikov et al., 2005; Kolaya and Firestein, 2021; Wu et al., 2021) and chronic (Golabchi et al., 2019) immune responses and damage repair processes, involving several distinct cell populations, such as erythrocytes, glial cells, neurons, etc. The processes include:

- Direct damage of the target tissue caused by the implantation of electrodes. The implantation of electrodes can mechanically cut or tear the target tissue, resulting in direct damage to local cells and blood vessels. This can lead to the entry of blood components (normally blocked by the blood–brain barrier) into brain tissue. Serum proteins, such as fibrinogen and albumin, adhere onto the surface of the electrode (Kolaya and Firestein, 2021). If the electrode materials of the HDMEAs are not biocompatible, they can cause local cell poisoning, irritation, and edema or even death. This, in turn, will further increase hydrostatic pressure around the electrode and harm more neurons, affecting the HDMEAs from collecting effective neuronal electrophysiological signals.
- Acute inflammatory reaction. The earliest inflammatory response can be caused by the erythrocytes, clotting, activated platelets, and blood vessels releasing factors due to vascular damage (Polikov et al., 2005; Wu et al., 2021). Serum proteins promote the activation of microglia and macrophages, which then cause inflammation near the electrode surface (Kolaya and Firestein, 2021). Microglia cells, one of the major glial cell types involved in wound healing response in brain, are activated after implantation and release a large number of chemicals, including neurotoxic factors such as chemokines, cytokines, reactive oxygen species, and neurotransmitters. Neurons near the electrode (about 100 μm around the electrode, affected by the implantation process) suffer from direct damage and injury from these cytokines or exogenous chemicals, thereby resulting in a decrease in neurons around the electrodes. As such malfunction of the electrode can happen among the surviving neurons (Kozai et al., 2015).
- Chronic body response. Chronic body response to HDMEAs is mediated by active astrocytes and activated microglia. After the early wound healing, activated microglia around the electrodes releases enzymes and reactive oxygen species to destroy external substances, which are eventually phagocytosed by microglia. Microglia also regulates the production of extracellular matrix (ECM) proteins which contribute to the formation of glial scars. Some external substances that cannot be cleared by microglia, such as implanted electrodes, will lead to further immune activation and glial cell proliferation. Ultimately, astrocytes proliferate and lead to the formation of glial scars around the electrode, preventing further damage. The presence of the probe in the tissue can trigger a continued foreign body response (Ward et al., 2009), especially when a mismatch exists between the electrode and tissue and micromovements happened (Karumbaiah et al., 2012; Polanco et al., 2016). Neurons lose their electrical activity after surviving from acute reaction (Abidian et al., 2010), resulting in reduced signal sources. The formation of glial scar can isolate the electrode from the surrounding neurons, reducing signal transmission by increasing the impedance of the tissue-electrode interface (Golabchi et al., 2019) and the distance between the electrode and target neurons (Rao et al., 2012).

## 4 Strategies and advances in developing next-generation flexible HDMEAs for closed-loop BMIs

### 4.1 Material advancement

Recent advancements in HDMEAs involve utilizing new materials for substrate and/or electroactive components or innovating with traditional materials to endow them with flexible properties. Compared to traditional materials, the newly developed materials possess unique properties specifically designed to address the demands of creating flexible HDMEAs. These properties include enhanced stretchability, better mechanical control, greater flexibility, and superior conductivity, among other advantageous characteristics. As a result, they are better equipped to tackle challenges in the mechanical, electrical, chemical, or biological domains. Some reviews by Gablech and Glowacki (2023) and Tringides and Mooney (2022) have been discussed about the material advances in MEAs development, thus we will just give an overview in this part.

To enhance biocompatibility, flexibility, and overall performance in neural interfaces, the advancement of substrate materials for flexible HDMEAs has incorporated innovative options such as hydrogels, silk, and polymers. Hydrogels, networks of hydrophilic, cross-linked polymer chains, have emerged as promising candidates for the next generation of bioelectronic interfaces due to their mechanical property similarity to biological tissue and versatility across electrical, mechanical, and bioengineering fields (Zhang and Khademhosseini, 2017; Park et al., 2021). Nevertheless, hydrogels also present challenges as substrate materials for flexible HDMEAs. Hydrogels’ low Young’s modulus can cause mechanical instability and deformation, their susceptibility to swelling and dehydration may alter interface stability, and their fabrication complexity restricts shaping for varied applications. By using the natural silk as an optical waveguide material, Zhou et al. (2022) presented and flexible opto-electro neural probe, in which electrode arrays of 128 recording channels were integrated on a single probe (Figure 4A). Silk has high transparency, excellent biocompatibility, and mechanical controllability. The Silk-Optrode probe, upon hydration of the silk optical fiber, autonomously adapts to its post-implantation environment, minimizing its mechanical stiffness to facilitate high-fidelity brain insertion while preserving mechanical compliance with adjacent tissue.

To improve the flexibility of the conductive parts, graphene (Sun et al., 2021), carbon nanotubes (CNTs) (Shih et al., 2020; He et al., 2021), and conducting polymers (CPs), such as PEDOT: PSS (Kshirsagar et al., 2019) have been developed for neural interfaces. Graphene, renowned for its notable flexibility and electrical conductivity, has become a pivotal material in neuronal interface studies and has been utilized to fabricate a range of flexible and stretchable electronic devices (Park et al., 2018). Still, it is vital to concurrently acknowledge and investigate its potential drawbacks, including long-term *in vivo* toxicity. Pure PEDOT: PSS hydrogels is brittle yet presents a high conductivity on the level of ≈ 40 S/cm. However, Li et al. (2022) created a highly conductive and stretchable double-network (DN) conducting polymer hydrogel from PEDOT: PSS and poly (vinyl alcohol; PVA) and achieved through *in situ* aggregation and densification, offering promising characteristics like high PEDOT: PSS content, electrochemical properties, and biocompatibility for potential use in bioelectronic applications (Figure 4B). Lee et al. (2022) developed flexible and transparent ultrathin (<10 nm) gold MEAs by using a biocompatible polyelectrolyte multilayer (PEM) metallic film nucleation-inducing seed layer (Figure 2C). With the polymer seed layer, the ultra-thin gold film created through thermal evaporation exhibits effective conductivity, along with high optical transparency and superior mechanical flexibility. Furthermore, liquid metals (notably eutectic gallium–indium, EGaIn) have garnered significant interest in the realm of stretchable biodevices (Dong et al., 2021), due to their exceptional mechanical attributes, electrical conductivity, and biocompatibility.

### 4.2 Design in geometries and shapes

The utilization of dual-side design has emerged as a vital strategy to increase the electrode density in flexible neural interface applications across several research papers. By employing a dual-side design, it is possible to enhance electrode density without enlarging the probe’s dimensions or reducing electrode size, thereby circumventing associated electrical challenges. Scholten et al. (2020) introduced a novel polymer-based microelectrode array with an impressive 512 platinum recording electrodes, optimized for chronic recordings in the brains of behaving rats, and showcase advancements in polymer microfabrication and back-side electrode patterning. In the work of Kim et al. (2023), a dual-side fabricated multimodal polymer neural probe was developed, featuring gold and platinum microelectrodes. Although a strategy of using multilayer with a sacrificial layer on the bottom could fabricate dual-sided flexible HDMEAs, it introduces challenges, such as misalignment during the multiple lithography processes (Liu et al., n.d.). A more straightforward and efficient method is still needed. Soft and stretchable electronics have been developed to create HDMEAs that can better conform to the brain’s surface and accommodate its natural movements. With some ingenious designs, these devices not only exhibit greater flexibility and stretchability but also reduce mechanical issues common in traditional designs. Moreover, due to improved contact between the electrode and tissue, they can ensure enhanced electrical performance. The stretchable opto-electric integrated neural interface (SOENI) is designed to align with the mouse brain’s somatomotor and somatosensory cortices (Ji et al., 2020a). It features a 3 × 3 microelectrode grid, 2 × 2 micro-LEDs, and a large reference electrode. Embedded within two silicone elastomer layers, the SOENI combines photostimulation and recording electrodes, with microscale light emitting diodes (LEDs) connected using a flip-on-chip method, aligning their illuminated surfaces with the microelectrode sites. Serpentine-shaped metal interconnecting wires were designed to improve the stretchability. Similar serpentine structures were also used in some other latest microsystems (Ji et al., 2020b; Xiang et al., 2022).

Mesh-like electrode designs have also been explored in order to improve the conformability of HDMEAs to the brain’s surface and minimize mechanical stress on cell or tissue. For example, Le Floch et al. (2022) reported “tissue-like” stretchable mesh nanoelectronics designed to align with brain organoids’ mechanical properties, which can be folded into 3D structures by progenitor or stem cells, facilitating three months electrophysiological measurements. Inspired by implantable mesh electronics and organoid polymer scaffold growth, McDonald et al. (2023) developed suspended mesh microelectrode arrays for neural organoids, incorporating four wells, each containing a mesh and 61 microelectrodes, which are adept for low-noise recordings and electrical stimulation with their sub-100 kΩ impedance at 1 kHz. These HDMEAs emphasized their ability to improve conformability to the brain’s surface and minimize mechanical stress. These designs represent a significant shift toward structures that are more adaptable and less invasive than traditional electrodes.

### 4.3 Surface modification strategies

Surface modification strategies not only address the challenges of electrical, mechanical, and biological compatibility but also significantly enhance the overall performance of HDMEAs in closed-loop BMIs. A common method of surface modification is adding a layer of special materials on the electrode surface to change the electrode’s properties. These materials include metals (Hempel et al., 2017), conductive polymers (Bianchi et al., 2021), nanostructures (Fattahi et al., 2014; Barai et al., 2018), and bioactive substances (Vitale et al., 2018; Golabchi et al., 2020). PEDOT and its derivative are used to improve the electrical properties (Hempel et al., 2017; Liu et al., n.d.) and modify the physical properties, such as transmittance (Yang et al., 2021b). The application of CNTs significantly increases the surface area, thereby increasing the charge storage capacity and the injection limit, with other benefits such as good adhesion, non-toxicity, and stable properties (Fattahi et al., 2014; Barai et al., 2018). Yang et al. (2023) used polypyrrole/carboxylated single-walled carbon nanotubes (PPy/SWCNTs) nanocomposites for electrode modification. The nanocomposites not only improved the performance of microelectrodes with low impedance (60.3 ± 28.8 kappa O) and small phase delay (−32.8 ± 4.4 degrees), but also presented stability for *in vivo* stimulation and recording for 21 days, as shown in Figure 3B. Neuroadhesive protein coating improves the chronic performance of neuroelectronics in the mouse brain (Golabchi et al., 2020).

Innovations in surface topology, with or without additional coating, have demonstrated improved recording quality compared to traditional planar electrodes. Mushroom-shaped microelectrodes improved the recording quality compared to planar MEA (Shmoel et al., 2016). The enhanced microscale wrinkles on microelectrodes were obtained by oil extraction from the elastic substrate and electroplating modified materials PEDOT: PSS and platinum black (Pt-black) on the wrinkled microelectrode sites improve the total device performance in electrocorticography (ECoG) signal recording without causing any cracks, delamination, or exfoliation (Shi et al., 2021).

### 4.4 Connection strategies for flexible HDMEAs

Anisotropic conductive film (ACF) bonding process has become a feasible approach to address the high-density interconnection challenges of flexible devices (Dagdeviren et al., 2014; Luo et al., 2023), yet, the high-temperature, high-pressure bonding process limits the use of some flexible materials. However, Hwang et al. (2021) introduced a stretchable anisotropic conductive film (S-ACF) capable of connecting high-resolution stretchable circuit lines to various electrodes, addressing the challenge of high-resolution stretchable interfacing at low temperatures through conductive microparticles in a thermoplastic film (Figure 3D).

Diverging from ACF, manufacturing a standard flat flexible cable (FFC) directly on the sensor to accommodate different connectors is also a viable approach. The zero insertion force (ZIF) connector can address the connection issue to some extent (Song et al., 2019; Kang et al., 2022), especially with its mechanical connection features, allowing sensors to be easily disconnected. This provides many conveniences for wearable, disposable HDMEAs. The easy-to-replace feature also makes it convenient for researchers to replace damaged sensors, which is especially valuable in some destructive experiments. However, this method seems a bit cumbersome when facing thousands of connection points. Especially the weight, size, and cost of the ZIF connector further restrict its application scenarios. The simplest method is to directly flip the sensor and affix the designed FFC directly onto a printed circuit board (PCB) or flexible PCB with conductive adhesive (Behfar et al., 2021). This method is straightforward, low-cost, and very suitable for HDMEAs with a smaller number of electrodes or in the prototype stage.

The instability of soft-hard interface can be addressed by either enhancing the adhesion between the materials or avoiding sudden changes from soft to hard, that is, by adding an intermediate layer/buffer layer to mitigate the transition. Ideally speaking, making the entire backend into flexible devices to form a fully flexible system would be best. By eliminating the differences between materials, this problem can be completely addressed. Zhang et al. report a capacitive pressure sensor (Zhang et al., 2022). Impressively, this sensor used CNT-doped PDMS, achieving both electrode and insulating materials using CNT-doped PDMS through tuning different doping concentrations. To some extent, this realized part of the full-flexibility goal. Such partial implementation alone resulted in surprisingly stable performance; the device remained stable even after 100,000 deformation cycles. This further confirms that aiming for a fully flexible design is undoubtedly a solution to the soft-hard interface issue.

### 4.5 Advances in fabrication techniques and strategies

Various recent fabricating strategies, including multi-layer strategies (Pimenta et al., 2021) and dual-sided micropatterning (Tooker et al., 2012; Pimenta et al., 2021; Kim et al., 2023), have been proposed to attain high-density probe capabilities without enlarging the polymer neural probes’ dimensions. Meanwhile, the advances in fabrication techniques also facilitate the development of flexible HDMEAs. Traditional fabrication techniques, such as photolithography, have significantly advanced the development of flexible HDMEAs, yet they come with limitations, including intricate processes, challenges in fabricating 3D structures, and precision constraints. Recently, emerging technologies, such as precise laser ablation and laser lithography, have introduced more streamlined, direct, and even more accurate methods for making complex electrode structures.

Laser ablation, or laser patterning, facilitates the streamlined fabrication of planar and 3D microelectrodes without the need for photolithography and etching by eschewing multi-step procedures for a more straightforward implementation (Tanwar et al., 2022). For example, soft and flexible gold microelectrodes with widths down to 3 μm were fabricated on a pliable PDMS substrate through a combination of supersonic cluster beam deposition and femtosecond laser processing (Dotan et al., 2021). Inkjet printing, garnering popularity for creating MEAs by directly depositing microelectrodes onto the substrate through single-step fabrication, facilitates the use of softer substrates compared to photolithography and permits the use of alternative conductive inks like PEDOT: PSS for printing conductive lines on soft substrate directly (Mandelli et al., 2021; Kim et al., 2022). However, it does come with the drawback of being notably expensive.

To effectively reduce crosstalk between interconnects, it is necessary to increase the distance or add a shield layer (Qiang et al., 2021; Naughton et al., 2022). Spacing them too far apart will inevitably increase the cable width and enlarge the overall system size. The core contradiction lies between the excessive number of wires and limited space. Some designs compensate for width by adjusting thickness, that is, by using multiple layers to reduce the number of wires that each single layer needs to accommodate (Shin et al., 2021; Saleh et al., 2022). Passive matrix and active matrix have become the mainstream architectures to address the signal readout of the array (Miccoli et al., 2019; Luo et al., 2023). The passive matrix design is relatively simple, consisting of intersecting rows and columns, with sensors set at the intersections (Sundaram et al., 2019; Kang et al., 2022). However, the crosstalk between sensors limits the quality of the signal. The active matrix addresses the crosstalk issue by adding switching units, such as diodes, to the electrodes (Park et al., 2019; Gwon et al., 2022). With the ideal performance comes complex circuit design requirements and internal structural issues. Nowadays, the flexible active matrix has become a mature technology used in the commercial display industry, which is worthy of designers’ study.

## 5 Conclusion

In conclusion, flexible HDMEAs have emerged as a promising technology for closed-loop BMIs, offering higher resolution, greater selectivity, and improved biocompatibility compared to traditional rigid electrodes. However, there are still many challenges that need to be overcome in the development and application of these arrays, such as mechanical and electrical stability, biocompatibility, power and energy efficiency, and clinical translation.

In the realm of future research directions for flexible HDMEAs, we identify several key research directions. The development of new materials is crucial, particularly for more ideal flexible substrates, conductive materials, and packaging solutions. These materials need to be stable, efficient, and durable for long-term use. Strategies for surface modification of the electrode are also essential, ensuring efficiency and stability over extended periods. Advances in fabrication, such as precise laser patterning and 3D printing, will greatly facilitate the creation of HDMEAs with complex structures and enhanced functionalities. In terms of connection technology, integrating wireless systems and remote monitoring will increase the clinical applicability of these devices. Investigating the use of flexible HDMEAs within closed-loop BMIs, and their application across a range of biological systems from rodents and humans to smaller organisms like insects, presents new opportunities in comprehending neural mechanisms, disease diagnosis and treatment, and biodetection.

## Author contributions

XL: Conceptualization, Methodology, Writing – original draft, Writing – review & editing. YG: Writing – original draft, Writing – review & editing. ZJ: Data curation, Writing – review & editing. TS: Data curation, Writing – original draft. WL: Conceptualization, Methodology, Resources, Supervision, Writing – original draft, Writing – review & editing.
